# Supplementary material for: Dynalign II: common secondary structure prediction for RNA homologs with domain insertions
Source: Nucleic Acids Res. 2014 Nov 21;42(22):13939–48. doi: 10.1093/nar/gku1172 (PMC4267632; doi:10.1093/nar/gku1172)
Supplement: SUPPLEMENTARY DATA [file supp_gku1172_nar-02021-z-2014-File012.zip › manual/Text/CircleCompare.html]

RNAstructure Command Line Help: CircleCompare


|  |  |  |
| --- | --- | --- |
|  | RNAstructure Command Line Help CircleCompare | - Contents - Index |
| CircleCompare is used to compare two nucleic acid structures for the same sequence by arranging them on top of one another around a circle. This layout makes base pairs and pseudoknotted pairs easily visible. USAGE: CircleCompare <predicted ct> <accepted ct> <ps file> [options] Required parameters: <predicted ct> \| The name of a CT file containing data for the predicted structure.; <accepted ct> \| The name of a CT file containing data for the accepted structure.; &ltoutput file> \| The name of an image file to which output will be written. Usually, this is a Postscript image file, although the user can specify that it be an SVG image file instead. To specify SVG images, the \"--svg\" flag must be specified in conjunction with a structure number flag (see the flags below). Options which don't require added values: -a, -A, --alternative \| Specify that an alternative color scheme should be used. CircleCompare uses the following color scheme when identifying secondary structure pairs: (Green \| Present in predicted and accepted structures; Red \| Present in predicted structure only; Black \| Present in accepted structure only) The alternative color scheme is similar to the default, but the colors used for accepted and predicted pairs differ: (Green \| Present in predicted and accepted structures; Purple \| Present in predicted structure only; Red \| Present in accepted structure only); -e, -E, --exact \| Specify exact comparison when structure comparison is scored. When exact comparison is specified, a known base pair between nucleotides *i* and *j* is considered to be correctly predicted only by the base pair *i*-*j*. By contrast, when flexible pairings are allowed, base pairs are considered to be correctly predicted when one of the two indices is different by up to one nucleotide. This better accounts for RNA dynamics and uncertainty in the RNA structure. For a known base pair *i*-*j*, all of the following predicted pairs are considered correct with flexible pairings: - *i* - *j* - *i* - *j*-1 - *i* - *j*+1 - *i*-1 - *j* - *i*+1 - *j* Default is to allow flexible pairings.; -f, -F, --file \| Specify that structure file names should be shown in addition to their descriptions. Default is not to show structure file names.; -h, -H, --help \| Display the usage details message.; -l, -L, --levorotatory \| Specify that the drawn structure is rendered counterclockwise. Default is to render drawn structures clockwise.; --svg \| Specify that the output file should be an SVG image file, rather than a Postscript image file. Note that only one SVG image can be written into a particular file, so the structure number flag (below) must also be specified when writing an SVG document.; -u, -U, --uncircled \| Specify that no circles should surround nucleotides when drawing. Default is to surround nucleotides with circles. Options which require added values: -n, -N, --number \| Specify the index of a particular structure in the predicted CT to be compared with the accepted CT, one-indexed. Default is -1, which signifies all structures output to one file.; -p, -P, --probability \| Specify the name of the file from which base pairing probability data will be read for annotation. The pairing data must be for the predicted structure. This file must be a partition function save file. Default is no probability annotation file used.; -p2, -P2, --probability2 \| Specify the name of the file from which base pairing probability data will be read for annotation. The pairing data must be for the accepted structure. This file must be a partition function save file. Default is no probability annotation file used.; -s, -S, --SHAPE \| Specify the name of the file from which SHAPE data will be read for annotation. This annotation is not structure dependent. Default is no SHAPE annotation file used.; -t, -T, --text \| Specify the name of the text file from which base pairing probability data will be read for annotation. This file should describe pairing data for the predicted structure, not the accepted structure. Default is no probability annotation file used. References: 1. Reuter, J.S. and Mathews, D.H. "RNAstructure: software for RNA secondary structure prediction and analysis." *BMC Bioinformatics*, 11:129. (2010). 2. Mathews, D.H., Sabina, J., Zuker, M. and Turner, D.H. "Expanded sequence dependence of thermodynamic parameters provides improved prediction of RNA secondary structure." *J. Mol. Biol.*, 288:911-940. (1999). | | |
| Visit The Mathews Lab RNAstructure Page for updates and latest information. | | |
